# Forced displacement and mental health problems in refugees residing in Quetta for decades

**DOI:** 10.1192/j.eurpsy.2023.640

**Published:** 2023-07-19

**Authors:** S. Sherzad, H. A. Khan, T. Sherzad

**Affiliations:** ^1^Psychiatry, Balochistan Institute of Psychiatry and behavioural Sciences; ^2^MBBS, Bolan Medical College, Quetta, Pakistan

## Abstract

**Introduction:**

As written by Warsan Shire, “no one leaves home until the home is the mouth of a shark”. UNHCR defines forced displacement as “displaced because of persecution, conflict, generalized violence or human rights violations"

**Objectives:**

to study the prevalence of common Mental health disorders among forcibly displaced people and comparing with the common mental health disorders among host community members.

**Methods:**

The OPD of BIPBS in SPH and BMCH of Quetta attends 800+ patients per month the data of the OPD of both hospitals was collected for Jan-May 2022 and was analyzed to numerate both the host community and refugees. out of 4120 for 354 refugee patients identified using their POR card and for 3776 of host community using their CNIC, data was analyzed for the prevalence of mental health disorders among them.

**Results:**

This study states that Afghan Refugees presented to OPD services of BIPBS, 47% were diagnosed as MDD with/without psychosis, 19% with GAD, 5% diagnosed as BAD, 5% With schizophrenia, 4% as PTSD, 3% as migraine, 3% conversion disorder, 2% OCD, 1% somatoform disorder and 10% of them presented with other psychiatric disorders, while in host community 21% were diagnosed as MDD with/without psychosis, 24% as GAD, 12% as somatoform disorder, 10% as OCD, 8% as migraine, 7% as conversion disorder, 4% as BAD, 3% as Schizophrenia, 3% as MBD due to substance misuse and rest of 7% presented with other psychiatric disorders.

**Conclusions:**

The conclusion of this study states that mental health disorders are more common among refugees than in other populations, the result of this study shows that there is a big difference in the prevalence of mental health disorders among displaced people and the rest of the population, some of the Mental health disorders are present in higher percentage among displaced people rather than among host community, while some other disorders are present in lower percentage among displaced people rather than among host community, this study also highlights that further studies are needed to determine, risk and protective factors within the host community.

**Disclosure of Interest:**

None Declared

